# Polio health economics: assessing the benefits and costs of polio, non-polio, and integrated activities of the Global Polio Eradication Initiative

**DOI:** 10.12688/gatesopenres.13524.1

**Published:** 2022-02-03

**Authors:** Kimberly M. Thompson, Dominika A. Kalkowska, Kamran Badizadegan

**Affiliations:** 1 Kid Risk, Inc, Orlando, FL, USA

**Keywords:** Polio, eradication, health economic, integration, cost-effectiveness analysis, benefit-cost analysis, gender equity

## Abstract

**Background**: Investments made by countries and donors to support polio eradication and the Global Polio Eradication Initiative (GPEI) over the past 35 years provided financial support for significant health interventions beyond the prevention of polio. Prior economic analyses that sought to quantify the economic benefits of some interventions encountered insufficient data and evidence associated with non-polio-specific activities. The 2022-2026 GPEI Strategic Plan explicitly identified integration and gender equity as funded mandates that must move forward in parallel with polio eradication, but these goals remain vaguely defined from a health economic perspective.

**Methods**: To ensure unambiguous and full accounting for all financial investments in the GPEI, polio eradication, and other desirable objectives, we identify the health economic analysis methods and inputs needed to ensure transparent financial accountability and cost-effective use of funds.

**Results**: Sufficient inputs and methods exist to characterize the health and economic benefits of polio-specific activities, but we identified the need for additional information and method development for some non-polio-specific and cost-sharing activities. Donors who seek to support non-polio-specific objectives as part of the GPEI may want to provide dedicated support financing for which it may be difficult to apply typical health economic criteria and to expect net health and/or net economic benefits.

**Conclusions**: Given the mixture of funding sources provided to the GPEI, which includes support by governments and private donors, we recommend that the GPEI separately account for financial needs that represent necessities for polio eradication from those used for other stated objectives. An added layer of specificity that identifies all funds according to each activity, the accountable party and/or parties, and the associated measurable health or other outcome(s), will enable improved health economic analyses and reporting to donors who seek to track returns on their investments.

## Introduction

Although several clinically significant diseases, including polio and measles, meet established international criteria for potential eradication ^1^, only a few have been targeted for eradication, and an even smaller number have been successfully eradicated. Thus, the potential to eradicate a disease does not necessarily mean that the global community will commit to its eradication, in large part because eradication not only requires significant financial resources, but also a global commitment to managing interdependent risks involving diverse and complex geopolitics. For example, the World Health Assembly (WHA) resolved in 1988 to eradicate polio by the year 2000 ^2^, and resolved in 1991 to eradicate dracunculiasis (guinea worm disease) by 1995 ^3^. In contrast, the WHA has not made similar resolutions for eradicating measles or rubella, despite the achievement of regional elimination of their indigenous transmission from the western hemisphere ^4, 5^ and stated commitments for measles elimination in all other regions of the world.

In addition, global experience with commitments to disease eradication and elimination remains mixed ^6^. Notably, in 1969, the WHA abandoned the 1955 commitment to eradicate malaria ^6^, in large part due to insufficient financial support and failures of national public health programs in many countries ^7^. Yaws eradication also failed ^1, 6, 8^ when “in the 1970s the vertical programmes in many countries were dismantled and yaws activities were integrated into the primary health care system to deal with the ‘last cases,’ [as a result of which] resources, attention and commitment for yaws activities gradually disappeared” ^9^. The yaws eradication experience of integrating activities provides an important signal for polio eradication efforts, which is also in the process of integrating resources and activities before achieving its primary goal of eradication ^10^. The loss of regional elimination status for measles in the western hemisphere (due to sustained transmission of imported measles) further demonstrates the challenges of maintaining elimination in some geographies, while other geographies remain endemic and capable of exporting the pathogen ^11^.

Eradication represents a major global public health project, which requires international commitment, cooperation, and substantial investments of infrastructure, human, technical, and financial resources, together with sustained political commitment ^1^. In theory, a WHA resolution to eradicate a pathogen or disease requires a real commitment from every country in the world to achieve the objective within its own borders using its national public health infrastructure and resources. In practice, however, many national public health systems lack sufficient resources and require financial and technical support from external sources. “A time-limited goal of eradication allows mobilization of support for a concentrated effort more readily than does a control program – both within countries where the disease is endemic and internationally. If developed countries have to spend resources to prevent or control importations of the disease (e.g., poliomyelitis, smallpox), such countries have additional incentive to help support an eradication campaign” ^1^. However, the dynamics of eradication ^12^ and the need for management of the globally interdependent risks posed by contagious agents that cross international borders require international institutions as well as highly committed global leadership and skilled diplomacy ^13^. For example, the creation of the World Health Organization (WHO) in 1948 provided a space within which national health officials could “conceive and articulate smallpox eradication as a global problem that required a coordinated global solution and then to pursue it as such” ^14^. Long-standing tensions between the need for eradication efforts to pursue “vertical” programs that provide the organization, strategy, management, financing, and accountability for performance required to obtain the goal (e.g., smallpox eradication), compete with “horizontal” programs that aim to support health services broadly (e.g., universal primary healthcare) ^14^.

The WHO has expanded its mission over time to move away from the management of globally interdependent health risks toward broader health and development goals ^15^. In reality, however, the priorities and the associated budgets set forth by the WHO Director General determine the WHO actions and its ability to manage the global threats posed by emerging or other infectious diseases of international concern (e.g., acquired immunodeficiency syndrome (AIDS) under Hafdan Mahler ^16^, and severe acute respiratory syndrome (SARS) under Gro Harlem Brundtland ^17^). The incentives for making and continuing commitments to global eradication and the required activities (e.g., immunization, surveillance, containment, and other risk management activities) will differ for stakeholders due to differences in their values and situations and the opportunities they see in the negotiations ^18, 19^. This reality creates opportunities for key leaders to hold the eradication activities “hostage to other concerns” (e.g., to secure funds for other (non-eradication) activities) in some cases ^18^. Committed leadership that prioritizes eradication and the management of interdependent risks at the highest level of international diplomacy (i.e., the United Nations) and that develops, manages, and holds itself accountable for a winning strategy represents a necessary, but not sufficient, prerequisite to successful eradication ^18^. As the global population size and mobility increases, preventing the establishment of emerging pathogens ^20, 21^ and eradicating established diseases becomes increasingly difficult and costly. For example, eradicating polio requires immunizing many more people than smallpox eradication required ^22^.

Despite the tensions between vertical and horizontal programs, vertical programs play a critical role in providing the surge capacity, infrastructure, and local community access needed to respond to emerging infectious diseases. Notably, vertical programs may find assisting with global health emergencies necessary to prevent disruption of their planned operations. For example, the Global Polio Eradication Initiative (GPEI) successfully helped to support the global response to the emergence of SARS in 2002–3 ^23^ and Ebola in 2014 ^24^, as well as other public health and humanitarian emergencies like the Asian tsunami of 2004 and floods in Pakistan in 2010–11 ^25^. In contrast, the GPEI did not play a preventive role with respect to the rapid emergence of SARS-CoV-2 in early 2020. However, following the declaration of the COVID-19 pandemic and the disruption of GPEI activities, the WHO fully utilized GPEI resources and infrastructure to manage the COVID-19 pandemic in countries in which GPEI maintained staff and equipment ^26^. From an economic perspective, unless the WHO reimburses the GPEI for the use of its resources, health economic analyses for the GPEI (e.g., 27) should include estimates of the COVID-19 and other health system benefits obtained through the use of GPEI resources during COVID-19. However, attributing the health and/or economic benefits will require full accounting of the GPEI resources used for COVID-19 response, and for other such emergency or incidental uses. For instance, total GPEI contributions to COVID-19 pandemic response efforts have already far exceeded the $26 million estimated for three WHO regions for January-June 2020 ^26^, but the full amount remains uncharacterized.

The 2022–2026 GPEI Strategic Plan explicitly identified desirable objectives (e.g., integration and gender equity) with scopes that go beyond the specific task of eradicating polioviruses ^10^. These activities are at various stages of programmatic development, but they remain relatively vague from a polio-specific health economic perspective. For instance, ensuring that all childhood immunizations remain intact after polio eradication is a critical global public health objective, but there is no polio-specific benefit from delivering measles and rubella vaccines in a polio-free world. To ensure economically efficient use of funds and complete accounting for all financial investments in polio eradication and associated non-polio GPEI investments, we identify the data requirements and health economic analysis inputs and methods needed to ensure financial accountability. This should help to support future GPEI-related health economic analyses (e.g., cost-effectiveness and benefit-cost analysis). We begin with a review of prior GPEI economic analyses and polio specific health economic analyses, and then turn to non-polio but GPEI-related topics.

## Prior polio economic analyses

Prior health economic analyses for polio eradication estimated substantial health and economic benefits ^28^. Despite the expected long-term financial benefits of polio eradication, prior to 2012 the GPEI developed short-term (e.g., annual) budgets based largely on anticipated contributions. The chronic funding gaps led to challenges ^27, 29– 31^, and this motivated multiyear planning and financing since the 2013–2018 GPEI Strategic Plan ^25^.

GPEI health economic analyses recognized some investments it made in non-polio activities, but faced considerable challenges quantifying the impacts ^27, 30^. A 2010 analysis estimated incremental net benefits associated with polio prevention of $42–47 billion (US$2008, equivalent to 52–59 billion US$2021, time-adjustments ^32^), if the GPEI succeeded in eradicating polio by 2012 ^30^. The analysis estimated additional benefits from vitamin A given during polio campaigns of $17–93 billion (US$2008, equivalent to 21–117 billion US$2021) ^30^. However, it highlighted the limited information available to quantify other health and financial benefits due to the use of GPEI infrastructure and capacity to assist in the international response to the emergence of SARS in the early 2000s ^23^).

With polio eradication still unfinished, an updated health economic analysis of the GPEI that assumed global conditions prior to the COVID-19 pandemic, estimated the incremental net polio-specific benefits on the order of 28 billion (US$2019, equivalent to 30 billion US$2021), if the GPEI succeeds in interrupting transmission by 2023 ^27^. The decline in expected incremental net benefits of the GPEI for polio prevention (i.e., from $52–59 billion to $30 billion in US$2021) reflects both the delay in achieving eradication, and the adoption of higher cost interventions (i.e., inactivated poliovirus vaccine (IPV) instead of and in addition to oral poliovirus vaccine (OPV)) ^27^. This analysis repeated mention of the insufficient information available to quantify the benefits and costs of non-polio activities supported by the GPEI ^27^, despite increasing recognition of the importance of GPEI spending on non-polio activities ^33^.

## GPEI transition and integration

As a mission-oriented enterprise, the GPEI recognized that it would end upon the achievement of eradication. The 2013–2018 GPEI Strategic Plan included a planning process to transition some of its activities that would need to continue longer term ^25, 34^. As described by the Polio Transition Independent Monitoring Board (TIMB) in 2021, for more than three decades, the GPEI infrastructure “has supported not only polio eradication-related activities, but also functions that go well beyond this core purpose, including: vaccine-preventable disease surveillance with the laboratory functions; essential immunization activities; new vaccine introductions in many countries; emergency preparedness and response; and health system strengthening” ^35^. The GPEI has also broadly cross-subsidized “operations support” for services such as “logistics, data, finance, human resources and administration” in many countries ^35^. As such, many countries in the “Africa, Eastern Mediterranean and South-East Asia Regions, have become heavily reliant on the GPEI infrastructure to sustain the broader public health functions” ^35^.

According to the first TIMB report in 2017, 25% to 50% of staff funded through the GPEI spent time on non-polio activities, and 95% of the polio assets were concentrated in 16 countries deemed most vulnerable to health system collapse with the loss of GPEI funding ^36^. The report estimated that the GPEI annual budget of nearly 1 billion US dollars provided vaccines to an estimated 430 million children and investigated 100,000 acute flaccid paralysis cases annually in a network of 145 laboratories spread across 92 countries. In support of this effort, GPEI contracted over 30,000 personnel and supported a much larger workforce, including volunteers and individuals paid on a daily or part-time basis ^36^. Polio eradication accounted for 20% (US$ 902.8 million) of the WHO annual budget in 2018–19 ^37^. 

Anticipating success of polio eradication by 2019, the WHO executive board prepared for substantial impacts on financial and personnel resources due to imminent loss of funding and closure of various GPEI functions ^38– 40^. The 2018 WHA adopted the Strategic Action Plan on Polio Transition ^33^, which identified the capacities and assets required to achieve the following three objectives: (1) maintain a polio-free world after eradication, (2) strengthen immunization systems and surveillance for vaccine preventable diseases in support of the WHO Global Vaccine Action Plan, and (3) strengthen emergency preparedness, detection and response capacity in countries to meet the WHO International Health Regulations (2005). This action plan ^33^ expanded on the polio-specific objectives identified by prior GPEI transition planning efforts ^41^, and specifically included non-polio related activities (objectives 2 and 3 above). The final report from a 2018 stakeholders’ meeting on polio transition reported the expected budget of US$ 667 million for the years 2019–2023 ^42^ for the WHO general programme of work (covering 2019, and the two subsequent WHO biennial budgets in 2020–2021 and 2022–2023). As part of polio transition, WHO is progressively shifting GPEI finances onto the WHO base financial ledger, which contrasts with the historical independent, parallel accounting and reporting. In a 2019 clarifying statement, WHO identified its accounting for polio transition funds as part of the WHO base budget, and stated that both the WHO and GPEI polio budgets “will be accounted for (base and non-base) to avoid the duplication and allow clarity and transparency of the budget and funding” ^43^. The WHO further indicated that “the GPEI budget subsumes both the base [polio transition] and non-base [polio eradication] portions of the WHO budget, since activities within the WHO base are also part of the overall GPEI budget and strategy” and that “the GPEI has committed to fundraise for all these resources” ^43^. The May 2021 (most recent) WHO proposed budget for 2022–23 ^44^ splits the budgets for polio eradication (US$ 558.3 million, non-base budget) and polio transition (US$ 322.1 million, base budget) into these two categories, consistent with the 2018 strategic action plan for polio transition. Notably, however, the 2022–2023 WHO budget ^44^ relied on multiyear GPEI budget estimates ^45^ from the 2019–2023 strategic plan issued prior to COVID-19 ^46^, which the GPEI will likely update soon for the 2022–2026 strategic plan ^10^. Since the WHO base budget approval by the WHA accommodates only relatively small deviations, and by design includes less flexibility than GPEI financing, the GPEI may need to create and maintain a contingency fund to manage transition-related financing challenges, including any overages implied by the GPEI expected financial resource requirements relative to the WHA-approved budget.

Overall, GPEI growth in the early 21st century led to a potential global leadership conflict of interest for WHO and others, because the loss of polio funding in countries or regions with weak public health infrastructures could lead to health system failures. GPEI assumed as part of its polio transition planning that: (1) to the extent possible, countries will absorb the costs of sustaining polio assets, (2) countries will map out the role polio assets play in their health systems and close any deficits created by the loss of polio funding, (3) national plans will align with the vaccination targets endorsed by WHO, and (4) donors will be prepared to fill any remaining gap ^36^. Despite these initial expectations, as of 2021, efforts to transition the responsibility for supporting all polio functions and activities to national governments remain incomplete based on process indicators tracked by the WHO monitoring and evaluation dashboard ^47^. Importantly, many countries have not yet assumed full responsibility for the costs of polio-related functions, which as the recent 2022–2026 GPEI Strategic Plan suggests, will lead to anticipated substantial increases in overall required financial resources for polio eradication ^10^ compared to prior expected GPEI annual budgets.

## Full accounting for GPEI and polio financial investments

### Investments in polio immunization and polio-specific eradication activities

The existing literature demonstrates the application of health economic analysis inputs and methods related to the use of poliovirus vaccines to prevent polio cases and reduce, eliminate, or eradicate poliovirus transmission ^28^. Thus, we find no need for additional development of inputs or methods to characterize the health and economic benefits of polio-specific GPEI or WHO activities (including poliovirus vaccines use to prevent polio).

With the emergence of COVID-19, speculations emerged about potential secondary vaccine effects (SVEs) ^48^ playing a role in fighting the pandemic, including some suggestions that OPV may offer secondary health benefits to protect against COVID-19 ^49^. Given the absence of specific, unbiased evidence of SVEs associated with poliovirus vaccines, however, health economic analyses for polio ^27, 50^ and for COVID-19 ^49^ did not include SVEs. A systematic review ^51^ found that the only published health economic analysis that quantified the use of OPV for potential SVEs did not find health or economic justification for repurposing OPV in the US for COVID-19 ^49^. Consistent with the findings of the analysis, the US did not reintroduce OPV for COVID-19 ^51^, and the development, licensure, and use of COVID-19 specific vaccines led to appropriate focus on widespread distribution of COVID-19 vaccines. In the absence of randomized controlled trials and demonstrated mechanisms of action that document specific effects of polio vaccines on non-polio health endpoints, health economic analysts lack sufficient inputs (e.g., costs, expected magnitudes and duration of effects) to support the inclusion of SVEs for poliovirus vaccines ^51^.

### Investments in non-polio-specific and cost-sharing activities

GPEI investments historically included subsidized and/or supported cost-sharing for some polio essential functions (e.g., surveillance, supply chain management). Polio assets (i.e., staff, equipment, and infrastructure that support polio essential functions) create capacity for performing other non-polio activities when the polio assets are not fully utilized for polio activities. In particular, the creation, expansion, and continued financial and technical support of the global polio laboratory network (GPLN) helped support the development of and remains a key component of global infectious disease surveillance ^52, 53^. As discussed above, GPEI transition planning recognized the key role of polio surveillance in global health, and WHO used GPEI assets to respond to public health emergencies including SARS, Ebola, and COVID-19. Full accounting of the benefits of GPEI investments will need to include characterization of the benefits of the use of polio-funded assets for management of public health emergencies and other diseases. 

The 2018 strategic action plan on polio transition ^33^ provided a framework for monitoring and evaluation of specific outcome indicators associated with objectives related to the use of polio assets for non-polio activities ( Table 1, left column). More recently, the WHO 2020 base budget ^44^ included currently tracked indicators for these same objectives, which WHO reports on using an online dashboard ( Table 1, right column) ^47^. Table 1 also shows evolution of the indicators over time. For example, the initial measles and rubella indicator evolved into three measles only indicators, and some other broad initial indicators dropped out. 

**Table 1.** WHO objectives and indicators related to the use of polio assets for polio-specific (objective 1) and non-polio-specific (objectives 2 and 3) activities as originally defined in Strategic Action Plan on Polio Transition (see references indicated in the table for the operational definition of each indicator).

Originally proposed indicators ^33, 54^	Currently tracked indicators ^44, 47^
*Objective 1: Sustain essential polio functions*	
• Inactivated polio vaccine coverage	• Coverage with inactivated poliovirus vaccine (IPV1) • Coverage with bivalent oral polio vaccine (bOPV)
• High-quality acute flaccid paralysis surveillance	• Number of cases of non-polio acute flaccid paralysis per 100,000 population aged less than 15 years • Percentage of acute flaccid paralysis case with adequate stool collection
• Polio event response	• Number of cases, type of poliovirus and duration of poliovirus outbreak • Number of sites and number of environmental surveillance samples per site
*Objective 2: Strengthen immunization systems*	
• Increased coverage with measles containing vaccine and rubella-containing vaccine	• Vaccine coverage with one dose of measles containing vaccine (MCV1) • Vaccine coverage with two doses of measles containing vaccine (MCV2) • Percentage of districts with MCV2 > 80%
• Countries with regular reporting of vaccine- preventable disease surveillance data from districts	N/A
• Expansion of surveillance and laboratory system at country level	N/A
• Government expenditure on routine immunization per newborn	• Government expenditure on routine immunization per newborn
*Objective 3: Strengthening emergency preparedness, detection and response capacity*	
• Health events detected and risk assessed early in health emergencies	N/A
• Populations affected by health emergencies have access to essential life-saving preventive and curative services and interventions	N/A
• Average value of the core capacity indicators of the International Health Regulations (IHR) (2005)	• Average % of IHR self-assessment annual reporting of laboratory core capacity • Average % of IHR self-assessment annual reporting of surveillance core capacity • Average % of IHR self-assessment annual reporting of emergency framework core capacity

The recent GPEI Polio Eradication Strategy 2022–2026 includes two polio-specific goals (i.e., permanently interrupting type 1 wild poliovirus transmission in Afghanistan and Pakistan, and stopping circulating vaccine-derived poliovirus (cVDPV) transmission and preventing outbreaks in non-endemic countries) ^10^. These two goals are in turn translated into five broad strategic objectives that include a mix of desired polio-specific and non-polio-specific outcomes, the latter primarily related to health system strengthening, integration, and gender equity ^10^. The strategic framework of mapping two polio-specific goals to five broad objectives that go beyond polio eradication was intentionally developed as a “holistic” tool that would result in “eradication through transformational and sustainable solutions.” The GPEI proposed to use a set of twenty seven key performance indicators (KPIs) to measure and monitor its success in achieving each of the five strategic objectives ^10^. The twenty seven KPIs include a set of six KPIs derived from the GPEI Gender Equality Strategy 2019–2023 ^55^, which specifically measure an outcome other than polio eradication. The remaining outcomes are not specifically divided into polio-specific and other indicators. Table 2 lists the KPIs that relate to non-polio-specific outcomes of integration, gender equity and/or health system strengthening. The working definitions for non-quantitative KPIs and the baseline status of the proposed KPIs are not currently available publicly.

**Table 2.** Non-polio-specific objectives and key performance indicators (KPIs) from the GPEI Polio Eradication Strategy 2022–2026 ^10^.

Objective	KPI
*Generate vaccine acceptance through* *context-adapted community engagement*	• % of female vaccinators per SIA in priority subnational areas in compliance with GPEI protection from sexual exploitation and abuse (PSEA) and safeguarding measures
	• Qualitative demonstration of the use of locally designed and implemented solutions informed by gender analysis to improve community engagement in polio and essential immunization campaigns
*Expedite progress towards eradicating polio* *and reducing zero-dose children through* *expanded integration efforts and unified* *partnerships*	• % of integrated service initiatives that are designed and implemented in a gender-responsive way
	• Increasing the amount to primary health care (PHC) investments directed towards polio high-risk areas in endemic and outbreak/at-risk countries
	• Continue to track polio human resource (HR) contributions towards COVID-19 response
*Improve frontline success through changes to* *campaign and outbreak response operations*	• % of campaigns for which microplans were developed via integrated planning workshops (inclusive of Expanded Programme on Immunization (EPI), maternal, newborn, child and adolescent health (MNCAH), communication and geographic information system (GIS)) in a gender-responsive way
*Improve detection and response through* *sensitive surveillance and containment*	• % of cases with adequate stool sample collection disaggregated by sex (target: 80% of cases)
*Gender Equality Strategy 2019–2023*	• % of GPEI interventions that are informed by gender analysis and that collect and analyze sex-disaggregated data
	• % of the GPEI 5-year budget allocation to gender mainstreaming
	• Existence of a policy on protection from sexual exploitation and abuse (PSEA) and safeguarding measures, and a workplan to implement the policy; staff member awareness of the existence of the policy on PSEA/ safeguarding and their appraisal of its effectiveness
	• % of staff trained and reporting increased knowledge levels, resulting in applying a gender perspective to their work
	• No. of women in decision-making roles at the headquarters (HQ), regional and country levels out of total no. of women
	• Perceptions of women and men in the GPEI on gender equality in decision- making

The performance indicators currently used by WHO ( Table 1) and proposed by GPEI ( Table 2) provide insights, but currently lack sufficient detail to support full economic evaluation of non-polio activities that receive polio eradication funds. Health economists will likely require both inputs and methods to fully value financial investments related to these non-polio objectives. Specifically, economists will want clarity in the accounting for the specific spending on gender equity (as a dedicated budgetary line item), and quantitative characterization of the changes in the gender equity obtained. Economists will also need to characterize the economic value of any changes. With respect to integration and health system strengthening beyond polio, economists will seek clarity about the definitions of activities that count as “integration” or “strengthening” and full accounting to capture any cost sharing with other programs and/or subsidization of polio activities by other programs. Economists will also need to characterize the economic value of any increase in the level of integration or the strength of global health system beyond that which is required to eradicate polio.

The WHO proposed budget for 2022–23 and specific indicators take steps in this direction ^44^. The specific indicators tracked by WHO may also provide a means for focusing analyses on specific non-polio health outcomes related to the interventions funded (or partially funded) by polio assets. These types of indicators should prove particularly helpful when they represent quantitative improvements that could feed directly into relevant models (e.g., for measles and rubella ^56^) for which some valuation inputs may already exist (e.g., for measles and rubella ^57^). For some other indicators (e.g., routine immunization per newborn, international health regulation (IHR) capacity), health economists will likely need to perform studies to develop quantitative valuation inputs to monetize the increases for these indicators. For indicators that require the development of valuation inputs, health economists will seek stable specific indicators that the WHO will report consistently over time, for which the established dashboard ^47^ should prove useful. Although economists would require further clarity about the sources of funds currently considered as part of the WHO base budget and potential overlap with GPEI budgeting, the reporting on polio as specific line items will support valuation. Ideally, the GPEI detailed budget for 2022–26 will also include the required level of specificity with respect to integration expenses that are not specifically related to the polio eradication program. 

For cost-sharing, economists will need to separate the polio-specific costs from the other activities, if those activities cover their part of the shared costs, and the health economic analysis would only count the associated polio health benefits. Alternatively, when polio or GPEI assets subsidize other activities, then full benefit and cost accounting would require economists to also estimate the health benefits of those other activities and to attribute an appropriate proportion of the gains to the polio or GPEI assets, or vice versa if non-polio activities subsidize polio activities. Any positive synergistic effects of integration (e.g., comprehensive vaccine preventable disease surveillance) and negative resource competition effects (e.g., insufficient funds leading to a failure to achieve and maintain standards for high-quality polio-specific surveillance) will require consideration. Complications may arise in the context of some non-polio activities performed with the intention of increasing polio immunization coverage (e.g., Polio Plus giveaways like soap, bed nets, etc.) if these provide health benefits that may prove difficult to quantify. However, studies that characterize cost functions for increasing coverage may provide a first approximation in the absence of better information ^58^.

## Discussion

Given the mixture of GPEI funding sources, which includes support by national governments (e.g., taxes collected) and private donor sources (e.g., gifts from high-net-worth individuals), we anticipate that separate accounting will be required to provide clarity for donors and enable better management of expectations. With respect to supporting health economic analyses, we recommend that the GPEI separately account for the financial needs required and resources spent for: (1) polio-specific immunization and eradication activities and (2) non-polio-specific activities, which should enable better attribution of costs and benefits and improved reporting to funding sources who seek to track performance on different specific objectives. To this end, we anticipate the need for donors who want to obtain specific non-polio objectives to provide dedicated financing for which they will not apply typical health economic criteria and/or not expect net health and/or economic benefits.

The recent emphasis on gender equity leads to a number of specific health economic research questions. With the GPEI specifying its expectation to allocate on the order of 1% of the total GPEI annual direct costs to gender equity, we hope that this will include the development of a depository of the specific interventions along with cost reporting and evaluations of effectiveness for specific interventions. As specific metrics for performance become established, the opportunity to perform contingent valuation studies that support the monetization of changes in the metrics may prove helpful. Some strategies to increase gender equity may not require any additional costs (e.g., preferentially hiring individuals of a specific gender at different levels to achieve the desired level of equity), whereas other strategies may imply real costs (e.g., increasing overall coverage to close a known gender gap in immunization coverage or surveillance). The importance of specification of the metrics for economic evaluations is essential for an objective like equity, because several strategies may exist to achieve the same result from an equity perspective (e.g., equal immunization coverage for males and females) that have different health or cost consequences (e.g., raising both to the same maximum level, lowering both to the same minimum, or equalizing at some level between the minimum and the maximum). 

Overall, characterization of the net health and economic benefits of investments in the GPEI and polio eradication became substantially more difficult under the new holistic strategic plan with the new emphasis on non-polio specific outcomes and integration. Health economists will therefore need increased transparency in the allocation of resources to different activities and full access to the cost and evaluation data. This transparency should also enable partners and donors to make better financial decisions by improving the characterization of the full costs and benefits of GPEI investments. With extended delays in achieving polio eradication eroding the expected polio-specific incremental net benefits of the GPEI ^27^, capturing the full benefits will become increasingly important to keep partners and donors engaged in the ultimate goal of polio eradication under the new strategic plan.

## Data availability

No data are associated with this article
