# Supplementary material for: Polio health economics: assessing the benefits and costs of polio, non-polio, and integrated activities of the Global Polio Eradication Initiative
Source: Gates Open Res. 2022 Feb 3;6:5. [Version 1] doi: 10.12688/gatesopenres.13524.1 (PMC8881365; doi:10.12688/gatesopenres.13524.1)
Supplement: Peer review history

# Peer review: Reviewer response for version 1

**DOI:** 10.21956/gatesopenres.14789.r31729

**Published:** 2022-02-25

**Authors:** Lisa Robinson

**Affiliations:** 1 Harvard T.H. Chan School of Public Health, Boston, MA, USA

This manuscript discusses the assessment of the benefits and costs of the Global Polio Eradication Initiative (GPEI). It provides information on the history of this initiative, summarizes previous economic analyses, discusses program transition and integration issues, lists program indicators, and argues for improved accounting methods that disaggregate funding necessary for polio eradication from funding used for other objectives. I provide some suggestions for the authors’ consideration below.

 1. Strengthen the organization and focus of the text: While the article provides an interesting survey of the issues, the connection between the details of the discussion and the points that the authors wish to make could be strengthened. The abstract provides a useful summary of the key points; more clearly structuring the text so it identifies each issue, walks through the evidence, and builds the links from the evidence to the conclusions would be helpful. Extraneous information that may be interesting but is not directly related to each point could be dropped.

 2. Clarify what is meant by “economic” analysis: This term covers a wide variety of methods, including conventional cost-effectiveness analysis and benefit-cost analysis, the use of wellbeing measures or social welfare functions, multi-criteria analysis, damage and burden assessment, budget impact analysis, etc. Each is designed to address different types of questions and has advantages and limitations. It is not clear which methods are being referenced here.

 Perhaps most importantly, conventional benefit-cost analysis focuses on estimating total social costs and benefits, within a specific normative framework (see reference below). If the key question relates to the link between financing and the outcomes supported, it is not clear that benefit-cost analysis alone would suffice.

 3. Recognize the need for methodological improvements. I was surprised by the statement on p.5 that “we find no need for additional development of inputs or methods to characterize the health and economic benefits of polio-specific GPEI or WHO activities (including poliovirus vaccines use to prevent polio).” Our work suggests that this is untrue. For example, there are significant gaps in the literature available for valuing mortality and morbidity risks, especially in low- and middle-income countries, leading to substantial uncertainty in the estimates. Key references include: Robinson *et al. *(2019 ^1^) and Robinson *et al. *(2019 ^2^).

Is the work clearly and accurately presented and does it cite the current literature?

Yes

If applicable, is the statistical analysis and its interpretation appropriate?

Not applicable

Are all the source data underlying the results available to ensure full reproducibility?

No source data required

Is the study design appropriate and is the work technically sound?

Yes

Are the conclusions drawn adequately supported by the results?

Yes

Are sufficient details of methods and analysis provided to allow replication by others?

Yes

Reviewer Expertise:

Benefit-cost analysis, health risk valuation, regulatory and policy analysis

I confirm that I have read this submission and believe that I have an appropriate level of expertise to confirm that it is of an acceptable scientific standard.

## References

[1] : Reference Case Guidelines for Benefit-Cost Analysis in Global Health and Development. *SSRN Electronic Journal* .2019; 10.2139/ssrn.4015886 10.2139/ssrn.4015886

[2] : Valuing Mortality Risk Reductions in Global Benefit-Cost Analysis. *J Benefit Cost Anal* .2019;10(Suppl 1) : 10.1017/bca.2018.26 15-50 10.1017/bca.2018.26 32968616PMC7473065

# Peer review: Reviewer response for version 1

**DOI:** 10.21956/gatesopenres.14789.r31727

**Published:** 2022-02-21

**Authors:** Brittany L. Hagedorn

**Affiliations:** 1 Bill and Melinda Gates Foundation, Seattle, WA, USA

Overall: This is an interesting and comprehensive overview of the state of tracking of GPEI finances.

 Additional clarity upfront on the goals of the review would be helpful, as I found myself unclear as to the hypothesis or research question that was being addressed. E.g. something to the effect of “we have identified a need for a review of the economic literature for X reasons, so we have done Y.” The authors may enjoy more readership if this clarity can be provided up front to motivate engagement with this extensive review.

 Abstract: The authors state that “Donors who seek to support non-polio-specific objectives as part of the GPEI may want to provide dedicated support financing,” but I wonder if this is truly possible as an objective. Yes, donors could fund specific line items, but since GPEI considers e.g. gender equity as a key part of their integrated strategy, it is not clear that these tasks could be funded in an optional way.  

 Intro: It would be good to have a citation for the following sentence, perhaps from budgets for non-polio elimination programs: “In practice, however, many national public health systems lack sufficient resources and require financial and technical support from external sources.”

 Intro: “As the global population size and mobility increases, preventing the establishment of emerging pathogens and eradicating established diseases becomes increasingly difficult and costly.” It would be great to provide additional clarity on why size and mobility drive difficulty and cost. You reference in the following sentence that the population is larger, but this is a really interesting point to expand on. Can the authors speak to other infectious disease dynamics here as well? This could include issues such as emergences due to environmental pressures, cross-border importations, sanitation challenges, etc.

 Intro: The authors argue that use of GPEI resources for COVID-19 response should be attributed to that disease and not accounted for in the cost of polio eradication. However, I am not convinced that this is necessarily required, since – as is pointed out in the introduction – political will is a key driver of successful eradication. One could argue that support for COVID-19 response is at a minimum a politically valuable choice that furthers buy-in from countries and WHO, and in doing so, supports polio eradication efforts.

 The authors make related arguments throughout the paper, and it would be good to address the long-term political value on such indirect work. For example, at the end of the intro it is stated that gender equity goes beyond the task of polio eradication, but one could argue that without addressing gender issues, GPEI will not be successful with eradication (e.g. if women cannot make choices about care seeking, this affects immunization rates). A more nuanced consideration of how this may have value and how it could be accounted for would be good to include.

 In the section “Investments in polio immunization and polio-specific eradication activities”, the combination of the two paragraphs is a bit disjointed and the heavy focus on COVID-19 SVEs seems like a mismatch to the header since it is not related to eradication activities. More generally, the COVID-19 section could be considered for removal, since that expenditure – if it had occurred – would not have been related to GPEI or eradication-related budgets and is thus not relevant for the broader goal of the manuscript.

 In the paragraph following table 2, the authors call for more detailed accounting of costs and inputs as they related to the non-polio-specific objectives. This makes sense generally. However, they also argue that there needs to be a way to account for the incremental benefits beyond what is ‘required to eradicate polio’, which implies that there is a threshold of improvement that could be identified that would be adequate to achieve eradication. It is not obvious that such as threshold is possible to define a priori, as we will only know what was required once eradication has been achieved. The authors should either explain in more detail why they think this is feasible or adjust the argument.

 Some of the content in the findings sections may be better suited to the discussion. For example, comments like “Ideally, the GPEI detailed budget for 2022–26 will also include the required level of specificity with respect to integration expenses that are not specifically related to the polio eradication program” and the last paragraph prior to the discussion. These are recommendations and thought exercises about the future, so should be shifted appropriately or rewritten for the findings section.

 In theory the call for increased accountability for non-polio spending makes sense, but I would like to see the authors provide an assessment of the potential downsides for doing so, in addition to the enumeration of the benefits. For example, calling for detailed accounting for gender equity related expenditures seems like it could generate large amounts of paperwork to track just 1% of the GPEI’s spending. There are both tangible costs (time and money to do the tracking) and intangible costs (e.g. bias to only doing tasks that can be counted) to doing this kind of detailed tracking, and this manuscript should include and address these concerns.

 The authors’ argument that there are several ways to achieve equity is valid, but perhaps there is a better demonstrative example than the one provided, since it seems unlikely that anyone would seriously propose achieving equity by lowering coverage.

Is the work clearly and accurately presented and does it cite the current literature?

Yes

If applicable, is the statistical analysis and its interpretation appropriate?

Not applicable

Are all the source data underlying the results available to ensure full reproducibility?

No source data required

Is the study design appropriate and is the work technically sound?

Yes

Are the conclusions drawn adequately supported by the results?

Yes

Are sufficient details of methods and analysis provided to allow replication by others?

Yes

Reviewer Expertise:

Cost effectiveness, budgetary analysis, health system strengthening.

I confirm that I have read this submission and believe that I have an appropriate level of expertise to confirm that it is of an acceptable scientific standard.
